# Aflatoxin B_1_, zearalenone and deoxynivalenol in feed ingredients and complete feed from different Province in China

**DOI:** 10.1186/s40104-016-0122-8

**Published:** 2016-10-22

**Authors:** Li Wu, Jianjun Li, Yunhu Li, Tiejun Li, Qinghua He, Yulong Tang, Hongnan Liu, Yongteng Su, Yulong Yin, Peng Liao

**Affiliations:** 1 Institute of Subtropical Agriculture, Chinese Academy of Sciences, 644# Yuandaer Road, Changsha, 410125 China; 2 ShenZhen University, Shenzhen, 518061 China; 3 NanJing Agriculture University, Nanjing, 210095 China; 4 JiangSu Aomai Bio-Technology Co., Ltd, Nanjing, 211226 China; 5 Hunan Biological and Electromechanical Polytechnic, The Party and Government Office, Donghu Road, Changsha, 410123 China

**Keywords:** Aflatoxin B_1_ (AFB_1_), Complete feed, Deoxynivalenol (DON), Feed ingredient, Zearalenone (ZEN)

## Abstract

**Background:**

The current study was carried out to provide a reference for monitory of aflatoxin B_1_ (AFB_1_), zearalenone (ZEN) and deoxynivalenol (DON) contamination in feed ingredients and complete feeds were collected from different Province in China from 2013 to 2015.

**Methods:**

A total of 443 feed ingredients, including 220 corn, 24 wheat, 24 domestic distillers dried grains with soluble (DDGS), 55 bran, 20 wheat shorts and red dog, 37 imported DDGS, 34 corn germ meal and 29 soybean meal as well as 127 complete feeds including 25 pig complete feed (powder), 90 pig complete feed (pellet), six duck complete feed and six cattle complete feed were randomly collected from different Province in China, respectively, by high-performance chromatography in combined with UV or fluorescence analysis.

**Results:**

The incidence rates of AFB_1_, ZEN and DON contamination of feed ingredients and complete feeds were 80.8, 92.3 and 93.9 %, respectively. The percentage of positive samples for DON ranged from 66.7 to 100 %. Domestic DDGS and imported DDGS presented the most serious contamination AFB_1_, ZEN and DON contamination levels of feeds ranged from 61.5 to 100 %, indicated that serious contamination over the studied 3-year period.

**Conclusion:**

The current data provide clear evidence that AFB_1_, ZEN and DON contamination of feed ingredients and complete feeds in different Province in China is serious and differs over past 3-year. The use of corn, domestic DDGS, imported DDGS and corn germ meal, which may be contaminated with these three mycotoxins, as animal feed may triggered a health risk for animal. Feeds are most contaminated with DON followed by ZEN and AFB_1_. Mycotoxins contamination in feed ingredients and complete feeds should be monitored routinely in China.

## Background

Mycotoxins are a large group of fungal secondary metabolites mainly produced from different fungal species in worldwide [1, 2]. Mycotoxin contamination is widespread among food and feed ingredients and is known to pose animal and human health risks in China, where aflatoxin B_1_ (AFB_1_), zearalenone (ZEN) and deoxynivalenol (DON) are prevalent [2, 3]. Among the document reported of more than 400 mycotoxins, and the most important mycotoxins in the contaminated feeds which is known to cause several adverse effects in pigs are AFB_1_, ZEA and DON, including negative effects on animal performance such as a decrease in feed intake and impairment of the immune system [3–11]. Considering that the gastrointestinal tract and the immune system of pigs are not vastly different that of humans, the pig can be regarded as a good model that can be applied to humans [4, 5]. Additionally, AFB_1_ is the potent of the known hepatotoxic, carcinogenic, mutagenic and teratogenic to animal and it usually triggered acute or chronic disease [4, 5]. Many published papers show the toxic effects of DON on animals, mainly impairing the immune system and the health status of the gastrointestinal tract and the brain [2, 4, 5, 7–11]. Ingestion of DON has also been associated with gastroenteritis, as reflected by nausea, emesis, diarrhea, anorexia and gastrointestinal hemorrhaging [4, 5, 11]. ZEN is an endocrine disruptor which it can be bind to estrogen receptor leading to reproductive function disorders and may have carcinogenic potential in humans [12].

Mycotoxins have a high toxicity for human and animal, several countries has been document maximum levels (MLs) and tolerance limits for these mycotoxins contamination in food or feed [1, 6]. For example, the MLs standard set of AFB_1_ for feed and completed feeding stuff from 5 to 20 μg/kg and maximum guidance levels of 250 μg/kg for ZEN and 900 μg/kg for DON in animal feeding products in the European Commission (Table 4) [3, 6, 13]. The government of China has renewed set tolerance limits for AFB_1_ range from 10 to 50 μg/kg in feed, their set MLs of ZEN is 500 μg/kg in complete feed, and their set the tolerance levels of DON from 1000 to 5000 μg/kg in complete feed (Table 4) [3, 6].

Nowadays, China faces a feed source shortage issue. Meanwhile, the high prices of protein source have leading to feed industrial use of alternative protein feed sources such as distillers dried grains with soluble (DDGS). In supplementation diet for DDGS for animal feed must be considered and detection of mycotoxins contaminationed, especially AFB_1_, ZEN and DON [14]. On the other hand, AFB_1_, ZEN and DON produce and fungal growth depend on climate environment such as high moisture and high temperature [3, 6, 14]. Corn, wheat and soybean are the main cash crops, and the potential hazards of the mycotoxin contamination of these cash crops have a greater impact. The Yangtze River basin and the Yellow River basin are one of the major grain producing areas of China. In this area, maize and wheat are the primary cash crops and are widely used in animal feed [15, 16]. Li et al. reported 50.0 % of maize samples collected from Beijing to be contaminated with AFB_1_ [3]. In another study in China, ZEN and DON were found in 96.6 and 93.2 % corn samples, with an average contamination level of 289.7 and 1356.9 μg/kg, respectively [14]. In the present study, a total of 443 feed ingredients were randomly collected from Jiangsu, Zhejiang, Shandong, Jiangxi, Inner Mongolia, Henan, Guangdong, Jilin, Anhui, Hebei, Sichuan, Shanxi and Fujian Province, and a total of 127 complete feeds were randomly collected from Jiangsu, Shandong, Henan, Zhejiang, Hebei and Anhui Province, respectively. Among of this regions, where the climate is warm and humid and there is plenty of rainfall, which is suitable for mould growth and mycotoxin formation. Ingram et al. reported that temperature and rainfall are the key climatic factors that influence plant pathogens and their secondary metabolites [17]. Therefore, mycotoxin contamination of feed ingredients is a serious problem in this region. Cheng et al. reported that in this regions, ZEN and DON contamination levels of wheat were 35.8 and 93.9 % in 2013, respectively [18]. Additionally, 93.75 % of maize was found to be contaminated by AFB_1_ in 2005 [19]. AFB_1_, DON and ZEN are largely prevalent in maize and wheat in this regions. Therefore, the contamination of feed materials by mycotoxin will result in increases in the mycotoxin levels of the feed products. The mycotoxins contamination in feed must be carefully monitored from different region and different years.

This study was conducted to detection the AFB_1_, ZEN and DON in feed contaminated from different Province in China. These results can serve as a reference for feed industrial, animal farm and China government regulatory of feed and food safety issue in the future.

## Methods

### Samples collection and preparation

The protocol for the analyses were reviewed and approved by the Institute Animal Care and Use Committed at Institute of Subtropical Agriculture, Chinese Academy of Sciences (Changsha, Hunan Province, China).

A total of 570 samples in our study were collected directly animal farms and animal production company from different location in China from 2013 to 2015. A total of 443 feed ingredient samples, including 220 corn, 24 wheat, 24 domestic DDGS, 55 bran, 20 wheat shorts and red dog, 37 imported DDGS, 34 corn germ meal and 29 soybean meal. A total of 127 completed samples, including 25 pig complete feed (powder), 90 pig complete feed (pellet), six duck complete feed and six cattle complete feed. All of the samples were undertaken according to the method by European Regulation No. 401/2006 [20]. All samples were grinded, mixed and stored at 4 °C until to analyses.

### High-performance liquid chromatography

AFB_1_ was analysis according to the previously methods [21]. Briefly, 20 g of ground feed was extracted with 100 mL of methanol:water (80:20, v/v), blended at high speed for 3 min and then filtered b Mycosep® #226 (Romer Labs. Inc., Singapore). The extract was diluted with a phosphate-buffered saline solution (PBS, pH 7.4), mixed well and filtered through microfiber filter paper. The immunoaffinity column (AokinImmunoClean CF AFLA, Aokin AG, Berlin, Germany) was conditioned with 1 mL of sodium azide, and 10 mL of the diluted filtrate was passed through the column at a flow rate of 1 mL/min. The column was then washed with 10 mL of a methanol: water solution (10:90, v/v) at a flow rate of 3 mL/min. The retained chemicals were then eluted with 1 mL of methanol at a flow rate of 1 mL/min. Subsequently, 20 μL of the clear eluate was injected directly into an HPLC system. If the eluate was found not to be clear, it was passed through an organic filter unit (0.45 μm) before injection. The mobile phase utilized a methanol:water solution (50:50, v/v) with the flow rate set at 1 mL/min. Post-column derivatization was performed with a photochemical reactor (AURA, Los Angeles, CA). A C_18_ column (4.6 mm × 250 mm, 5 μm, Dikma, Shanghai, China) was employed with the LOD set at 0.5 ppb and the LOQ at 1.5 ppb. A fluorescence detector (SHIMADZU, Kyoto, Japan) was set for excitation and emission wavelengths of 360 and 440 nm, respectively. The retention time was 16.5 min. The temperature of the column was set for 30 °C.

ZEN and DON analysis according to the methods of GB/T 23504-2009 and GB/T 23503-2009 [6, 14]. For ZEN analysis, samples were analyzed according to the certified Chinese GB/T 23504–2009 method. Briefly, 25 g of feed was extracted with 100 mL of a methanol:water solution (60:40, v/v), blended at high speed for 3 min and then filtered through a sheet of Waterman Filter Paper No. 4. The extract was diluted with a phosphate-buffered saline solution (PBS, pH 7.40), mixed well and filtered through microfiber filter paper. The immunoaffinity column (AokinImmuno-Clean CF ZEA, Aokin AG, Berlin, Germany) was conditioned with 1 mL of sodium azide, and 10 mL of the diluted filtrate were passed through the column by gravity at a flow rate of 1 mL/min. The column was then washed with 10 mL of a methanol:water solution (10:90, v/v) at a flow rate of 3 mL/min. The bound chemicals were then eluted with 3 mL of methanol at a flow rate of 1 mL/min. Subsequently, 20 μL of the clear eluate was injected directly into an HPLC system. If the eluate was found not to be clear, it was passed through an organic filter unit (0.45 μm) before injection. The mobile phase utilized an acetonitrile:water: methanol solution (46: 46: 8, v/v/v with the flow rate set at 1 mL/min. A C_18_ column (4.6 mm × 150 mm, 5 μm, Dikma, Beijing, China) was employed with the LOD set at 1.5 ppb and the LOQ at 4 ppb. A fluorescence detector (SHIMADZU, Kyoto, Japan) was set for excitation and emission wavelengths of 274 and 440 nm, respectively. The retention time was 7.3 min.

For DON analysis, samples were analyzed according to the method of GB/T 23503–2009. Briefly, 25 g of ground feed was extracted with 100 mL of a solution of methanol:water (60:40, v/v), blended at high speed for 3 min and then filtered through a sheet of Waterman Filter Paper No. 4. The extract was diluted with a phosphate-buffered saline solution (PBS, pH 7.4), mixed well and filtered through microfiber filter paper. The immunoaffinity column (AokinImmunoClean CF DON, Aokin AG, Berlin. Germany) was conditioned with 1 mL of sodium azide, and 10 mL of the diluted filtrate was passed through the column by gravity at a flow rate of 1 mL/min. The column was then washed with a 10 mL solution of methanol:water (10:90, v/v) at a flow rate of 3 mL/min. The bound compounds were then eluted with 3 mL of methanol at a flow rate of 1 mL/min. The purified samples were dried under a stream of nitrogen gas at 50 °C. For the mobile phase, the residue was dissolved in 200 μL after evaporation. Subsequently, 20 μL of the eluate was injected into an HPLC system. The mobile phase utilized a methanol: water solution (30: 70, v/v) with the flow rate set at 1 mL/min. A C_18_ column (4.6 mm × 150 mm, 5 μm, Agilent, Santa Clara, CA) was employed with the LOD set at 0.02 ppm and the LOQ at 0.06 ppm. The absorption UV wavelength (SHIMADZU, Kyoto, Japan) was set at 218 nm. The retention time was 5.6 min.

### Data analysis

All data were calculated using Microsoft Excel 2007 and are expressed as percentages or means, median and maximum.

## Results and discussion

### Mycotoxin occurrence

All of AFB_1_, ZEN and DON occurrence data are showed in Tables 3 and 4. In summary, detected feed ingredients and complete feeds were mostly contaminated by DON, followed by ZEN and AFB_1_. A total of 573 samples including 443 samples of feed ingredients and 130 samples complete feeds were analyzed to determination AFB_1_, ZEN and DON. Of these samples analyze for DON, 93.9 % contained this mycotoxin, with levels ranging from 0 to 4402.7 μg/kg. The occurrence rate of ZEN and AFB_1_ were 92.3 and 80.8 %, respectively.

### AFB_1_ in feed ingredients and complete feeds

The results of AFB_1_ were shown in Table 1 from 2013 to 2015. AFB_1_ was analysis in corn samples in 2013, with levels ranging from 0 to 15.9 μg/kg, and in 67.6 and 82.4 % of the corn samples in 2013 and 2015, respectively. From 2013 to 2015, all of the corn samples were not exceeding to 50.0 μg/kg regulatory limits in China. Moreover, in 2005, Gao et al. analysis 279 corn samples collected in China and found AFB_1_ contaminated in 74.6 % in detection samples, with an average value for 39.64 μg/kg [19]. A similar study was shown in Bhat et al. document, he analysis 2074 samples of corn collected from rural and urban areas of 11 states for AFB_1_ contamination and analysis a median value from <5 to 35 μg/kg [22]. The prevalence of AFB_1_ contamination in wheat was almost the same in the 3 year investigated and was significantly lower than that observed in corn. The domestic DDGS and imported DDGS were more seriously contaminated by AFB_1_ than bran and wheat shorts and red dog. It was highly contaminated in 2013 and 2014, with 100 and 100 % of the domestic DDGS and imported DDGS samples contaminated by AFB_1_, respectively. AFB_1_ detection results showed that it’s to exceeded regulatory limits, with an average levels of 10.0 (Table 4). AFB_1_ detection rate and concentrations were higher than the previously study by Li et al. [3]. AFB_1_ contaminated in corn germ meal and soybean meal results were lower than those found in the corn, domestic DDGS and imported DDGS and did exceeding the maximum limits set in China (Table 4). Li et al. document results showed that 6 % of the DDGS contained concentrations of AFB_1_ that exceeded regulatory limits, with an average content of 9.8 μg/kg in 2011. From 2014 to 2015, all of the domestic DDGS samples were exceeding to 9.8 μg/kg regulatory limits in China, with an average content of 10.2 and 10.9 μg/kg. From 2014 to 2015, all of the imported DDGS samples were exceeding to 9.8 μg/kg regulatory limits in China, with an average content of 10.4 and 10.0 μg/kg, respectively. These result was not agreement with the results of a survey in China occurrence of mycotoxins in feedstuff and feed conducted by Li et al. [3]. Possible reason was that feed mill and farmers pay more attention to storage conditions because of the highly prices of soybean meal as a protein source feed.

**Table 1:** Analyses of AFB_1_(μg/kg) in feed ingredients and complete feeds

Item	Growing years	Numbers of samples	Positive samples				Numbers of samples in the range, μg/kg				
			%^a^	Mean	Median	Maximum	<0.5	0.5–10	10–50	50–500	>500
Feed ingredients											
Corn	2013	74	67.6	3.5	1.4	15.9	24	44	6	0	0
	2014	55	94.6	4.3	3.7	11.1	3	50	2	0	0
	2015	91	82.4	3.9	4.4	25.5	16	73	2	0	0
Wheat	2013	11	27.2	0.4	0	1.8	8	3	0	0	0
	2014	8	62.5	1.1	1.0	4.0	3	5	0	0	0
	2015	5	80.0	1.5	1.3	3.0	1	4	0	0	0
Domestic DDGS	2013	6	100	10.5	9.7	13.1	0	4	2	0	0
	2014	11	100	10.0	10.2	13.6	0	3	8	0	0
	2015	7	100	11.0	10.9	12.7	0	1	6	0	0
Bran	2013	27	59.3	3.1	1.3	10.9	11	14	2	0	0
	2014	10	90	2.4	2.3	4.5	1	9	0	0	0
	2015	18	83.3	2.1	2.4	3.8	3	15	0	0	0
Wheat shorts and red dog	2013	7	100	6.0	8.1	9.3	0	7	0	0	0
	2014	3	100	4.3	1.5	10.5	0	2	1	0	0
	2015	10	80	5.0	6.2	8.0	2	8	0	0	0
Imported DDGS	2013	7	100	9.0	9.1	13.7	0	4	3	0	0
	2014	17	100	10.5	10.4	15.2	0	7	10	0	0
	2015	13	61.5	7.0	10.0	13.8	5	2	6	0	0
Corn germ meal	2013	6	83.3	4.2	1.8	10.2	1	3	2	0	0
	2014	9	88.9	9.3	10.6	13.5	1	2	6	0	0
	2015	19	68.4	7.5	10.2	14.1	6	3	10	0	0
Soybean meal	2013	13	92.3	4.5	3.1	9.8	1	12	0	0	0
	2014	3	100	6.4	6.4	7.1	0	3	0	0	0
	2015	13	84.6	2.6	2.2	6.2	2	11	0	0	0
Total	-	443	-	-	-	-	-	-	-	-	-
Complete feeds											
Pig complete feed (powder)	2013	10	90	6.4	7.9	5.4	1	8	1	0	0
	2014	2	100	6.3	7.9	5.2	0	2	0	0	0
	2015	13	100	19.9	8.2	9.1	0	13	0	0	0
Pig complete feed (pellet)	2013	19	68.4	4.6	5.2	11.1	6	13	0	0	0
	2014	33	100	8.0	7.3	18.1	0	26	7	0	0
	2015	38	63.2	3.4	2.5	9.6	14	23	1	0	0
Duck complete feed	2013	-	-	-	-	-	-	-	-	-	-
	2014	-	-	-	-	-	-	-	-	-	-
	2015	6	100	6.44	3.97	8.84	0	6	0	0	0
Cattle complete feed	2013	-	-	-	-	-	-	-	-	-	-
	2014	-	-	-	-	-	-	-	-	-	-
	2015	6	100	4.5	3.9	8.3	0	6	0	0	0
Total	-	127	-	-	-	-	-	-	-	-	-

^a^Positive samples are defined as those with aflatxoin B_1_ ≥ 0.5 μg/kg (LOD)

AFB_1_ detection results in complete feed samples were shown in Table 1. The AFB_1_ contamination in complete feed increased from 2013 to 2015. Pig completed feed (powder) and pig complete feed (pellet) contamination value were significantly higher those in duck and cattle complete feed in our study. Pig complete feed (powder) and pig complete feed (pellet) were the feed with most serious contamination by AFB_1_, with value ranging from 63.2 to 100 %, followed by duck complete feed (50–100 %) and cattle complete feed (0–100 %). These results not agreement with the maximum and average values reported by previously survey [3, 6, 14]. A possibility explanation for discrepancies between previously study and our present study could be that the different climate environment and detection methods for collection samples.

### ZEN in feed ingredients and complete feeds

Over the studied 3-years period, the ZEN contamination levels of corn, wheat, domestic DDGS, bran, wheat shorts and red dog, imported DDGS, corn germ meal, soybean meal and complete feeds (except for cattle complete feed) were greater than 50 % and mean values detection for corn, wheat, domestic DDGS, bran, wheat shorts and red dog, imported DDGS, corn germ meal, soybean meal, pig complete feed(including powder and pellet) were exceeded 150 μg/kg in 2013 to 2015 (Table 2). In 2015, 99 % of the corn samples were contaminated, with levels ranging from 0 to 1442.5 μg/kg and the maximum level (1442.5 μg/kg) was high than those detectioned in 2013 (780.2 μg/kg) and 2014(833.9 μg/kg). Meanwhile, in 2015, 89.5 % of the corn germ meal samples were contaminated, with levels ranging from 0 to 1518.2 μg/kg and the maximum level (1518.2 μg/kg) was high than those detectioned in 2013 (581.1 μg/kg) and 2014 (582.1 μg/kg). Additionally, the highest ZEN contaminated levels of pig complete feed (pellet) occurred in 2015 (94.7 %), but the highest maximum (1296.5 μg/kg), average (375.0 μg/kg) and median value (283.2 μg/kg) in 2015. Rodrigues et al. showed contaminated percentages and ZEN values in corn samples from Middle East and African countries of 16 % (maximum 310 μg/kg) [23]. Li et al. reported for central China 84.1 % ZEN contaminated in corn samples at values ranging from 10.1 to 1613.7 μg/kg [3]. Another detection study in China, ZEN was found in 96.6 % of corn samples, with an average contamination values of 289.7 μg/kg to 1894 μg/kg [14]. Most of survey findings are consist with our study results, showing the seriousness of ZEN contamination in corn and pig complete feed, it must be more carefully used in feed mill.

**Table 2:** Analyses of ZEN (μg/kg) in feed ingredients and complete feeds

Item	Growing years	Numbers of samples	Positive samples				Numbers of samples in the range, μg/kg				
			%^a^	Mean	Median	Maximum	<10	10–100	100–500	500–2000	>2000
Feed ingredients											
Corn	2013	74	91.9	236.7	236.2	708.2	6	2	65	1	0
	2014	55	96.4	223.5	215.8	833.9	2	1	51	1	0
	2015	91	99	279.1	257.1	1442.5	1	5	77	8	0
Wheat	2013	11	91	110.5	133.3	152.1	1	3	7	0	0
	2014	8	87.5	128.56	146.075	161.8	1	0	7	0	0
	2015	5	100	127.9	135.5	156.3	0	1	4	0	0
Domestic DDGS	2013	6	100	388.5	381.1	480.8	0	0	6	0	0
	2014	11	100	290.9	332.05	371.5	0	0	11	0	0
	2015	7	100	416.4	420.9	529.6	0	0	5	2	0
Bran	2013	27	100	139.8	126.7	329.0	0	4	23	0	0
	2014	10	100	166	161.81	246.9	0	1	9	0	0
	2015	18	94.4	150.7	144.6	293.6	1	3	14	0	0
Wheat shorts and red dog	2013	7	100	219.3	225.0	280.3	0	0	7	0	0
	2014	3	100	209.4	265.6	276.0	0	1	2	0	0
	2015	10	100	199.0	213.4	275.7	0	0	10	0	0
Imported DDGS	2013	7	85.7	273.7	301.8	444.6	1	0	6	0	0
	2014	17	94.1	313.4	323.2	419.0	1	0	16	0	0
	2015	13	100	363.7	349.8	510.3	0	0	12	1	0
Corn germ meal	2013	6	83.3	342.8	371.7	581.1	1	0	4	1	0
	2014	9	77.8	278.1	317.6	582.1	2	0	6	1	0
	2015	19	89.5	630.2	630.3	1518.2	2	0	8	9	0
Soybean meal	2013	13	100	206.6	224.2	325.7	0	0	13	0	0
	2014	3	66.7	149.8	215.8	233.6	1	0	2	0	0
	2015	13	100	178.6	194.5	332.5	0	2	11	0	0
Total	-	443	-	-	-	-	-	-	-	-	-
Complete feeds											
Pig complete feed (powder)	2013	10	90	214.7	225.9	455.8	1	2	7	0	0
	2014	2	100	253.0	253.0	287.1	0	0	2	0	0
	2015	13	100	348.6	295.5	835.4	0	0	11	2	0
Pig complete feed (pellet)	2013	19	84.2	232.5	253.3	435.8	3	0	16	0	0
	2014	33	66.7	197.6	214.5	862.4	11	1	19	2	0
	2015	38	94.7	375.0	283.2	1296.5	2	0	27	9	0
Duck complete feed	2013	-	-	-	-	-	-	-	-	-	-
	2014	1	-	-	-	-	-	-	-	-	-
	2015	6	100	307.0	303.8	357.9	0	0	6	0	0
Cattle complete feed	2013	-	-	-	-	-	-	-	-	-	-
	2014	-	-	-	-	-	-	-	-	-	-
	2015	6	-	0	0	0	0	0	0	0	0
Total	-	127	-	-	-	-	-	-	-	-	-

^a^Positive samples are defined as those with zearalenone ≥ 10 μg/kg (LOD)

### DON in feed ingredients and complete feeds

The detection results of DON in feed ingredient and complete feeds are shown in Table 3. In 2014 and 2015, corn, bran, imported DDGS, pig complete feed (including powder and pellet) and duck complete feed were highly contaminated with DON, as maximum, average and median values occurred. Corn, cron germ meal and pig complete feed (pellet) present the highest contamination rate with the percentage of DON-contaminated corn germ meal and pig complete feed (pellet) samples were 100, 89.5 and 84.2 % in 2015, whereas DON was detectioned in only 88.9 and 66.7 % of corn germ meal and pig complete feed (pellet) samples in 2014, respectively. All of results in our study showed that DON contamination values are first to those of ZEN. Guan et al. showed that 93.2 % of corn sample collected from China in 2009 were contaminated with DON, with average values of 1357 μg/kg and maximum levels of 5150 μg/kg. Meanwhile, Binder et al showed that 70 % of corn samples in Asia were contaminated with DON, with a maximum values of 10,626 μg/kg, which is similar to that measured in Li et al. study [3, 24]. Moreover, Cui et al. showed that DON was found in 89.3 % of wheat samples harvested from Jiangsu and Anhui Province at values ranging from 259 to 4957 μg/kg, which is consist with our study results (4402.7 μg/kg in corn germ meal) [25]. Corn is an energy ingredient frequently used in animal feeds in China, in the years in which corn and its by-products were found to be highly contaminated with DON and ZEN, the corresponding analysis rate in pig complete feed were high. More interesting, in 2015, the ZEN contamination in corn, corn germ meal and pig complete feed (including powder and pellet) were significant highly.

**Table 3:** Analyses of DON (μg/kg) in feed ingredients and complete feeds

Item	Growing years	Numbers of samples	Positive samples				Numbers of samples in the range, μg/kg			
			%^a^	Mean	Median	Maximum	<100	100–1000	1000–5000	>5000
Feed ingredients										
Corn	2013	74	96.0	525.6	552.4	1271.5	3	70	1	0
	2014	55	98.2	603.3	382.9	4320.9	1	48	6	0
	2015	91	100	1024.2	738.2	3625.3	0	51	40	0
Wheat	2013	11	100	627.8	615.8	1048.1	0	10	1	0
	2014	8	100	774.6	774.6	1048.1	0	7	1	0
	2015	5	100	485.7	537.6	660.1	0	5	0	0
Domestic DDGS	2013	6	83.3	1032.1	925.4	1997.6	1	3	2	0
	2014	11	100	1147.8	1101.9	1492.4	0	4	7	0
	2015	7	100	1794.5	1864.0	2146.8	0	0	7	0
Bran	2013	27	96.3	839.7	867.5	1431.3	1	21	5	0
	2014	10	100	1142.4	980.7	3503.2	0	8	2	0
	2015	18	100	1005.9	864.3	2532.5	0	12	6	0
Wheat shorts and red dog	2013	7	100	505.4	410.3	1030.2	0	6	1	0
	2014	3	100	434.9	466.9	595.4	0	3	0	0
	2015	10	100	659.8	613.9	1319.5	0	9	1	0
Imported DDGS	2013	7	100	1079.6	774.3	1766.6	0	4	3	0
	2014	17	94.1	1088.9	1063.11	1875.1	1	6	10	0
	2015	13	100	2186.8	2033.23	3561.0	0	0	13	0
Corn germ meal	2013	6	100	854.2	975.5	1026.6	0	3	3	0
	2014	9	88.9	1017.3	868.4	1989.2	1	5	3	0
	2015	19	89.5	2045.5	1255.1	4402.7	2	3	14	0
Soybean meal	2013	13	100	567.2	566.0	786.4	0	13	0	0
	2014	3	66.7	348.5	489.3	556.1	1	2	0	0
	2015	13	100	364.5	331.2	662.9	0	13	0	0
Total	-	443	-	-	-	-	-	-	-	-
Complete feeds										
Pig complete feed (powder)	2013	10	90	791.3	804.6	1602.6	1	8	1	0
	2014	2	100	523.4	523.4	623.6	0	2	0	0
	2015	13	100	1216.3	1065.9	2767.6	0	5	8	0
Pig complete feed (pellet)	2013	19	100	660.3	719.2	946.6	0	19	0	0
	2014	33	66.7	537.7	450.2	2478.3	11	19	3	0
	2015	38	84.2	704.0	631.4	3346.0	6	17	15	0
Duck complete feed	2013	-	-	-	-	-	-	-	-	-
	2014	-	-	-	-	-	-	-	-	-
	2015	6	100	1718.3	1846.2	2613.7	0	6	0	0
Cattle complete feed	2013	-								
	2014	-								
	2015	6	0	0	0	0	0	0	0	0
Total	-	127	-	-	-	-	-	-	-	-

^a^Positive samples are defined as those with deoxynivalenol ≥ 100 μg/kg (LOD)

Contamination with AFB_1_, DON and ZEN was particularly serious in domestic DDGS and imported DDGS samples. In our study, all of the domestic DDGS and imported DDGS samples contamination with AFB_1_, DON and ZEN were higher than the previously survey data in China [6, 16, 26]. The percentage of samples containing concentrations of DON that exceeded regulatory limits and the average content of DON detected in domestic DDGS and imported DDGS samples were higher than the concentrations of DON that have previously been found in distillers dried grains with soluble sourced worldwide [3, 27]. These results may be explained by the fact that mycotoxins in DDGS (constituting the remaining portions within the final by-product) are up to three times more concentrated than in corn grain [28]. Furthermore, if improperly stored, DDGS are easily contaminated with more mycotoxins due to their high moisture content. In addition, grains are damaged during the process of DDGS production, which can easilym cause the production of more mycotoxins. Moreover, in our study, the domestic DDGS and imported DDGS were obtained from the regions lying along the middle and lower reaches of the Yangtze River (in Henan, Sichuan, Anhui and Jiangsu provinces) which are characterized by a moist climate which may contribute to the contamination of corn with mycotoxins [16, 26]. What is more, this study has shown that multiple mycotoxins coexist in most feeds and feedstuffs [24, 29]. About 29 % (5/17) of the DDGS samples used in this study were co-contaminated with DON and ZEN at levels of 1 ppm and 500 ppb, respectively, which exceeds Chinese regulatory limits for feedstuffs. The simultaneous occurrence of contamination by various kinds of mycotoxins leads not only to immune suppression in animals, but also lowered efficiency in animal production [30].

The natural mycotoxins contamination has been reported in variety of foods and feeds in worldwide. AFB_1_, ZEN and DON production and fungal growth are independent on climate environment conditions. In the present study, a total of 440 feed ingredients were randomly collected from Jiangsu, Zhejiang, Shandong, Jiangxi, Inner Mongolia, Henan, Guangdong, Jilin, Anhui, Hebei, Sichuan, Shanxi and Fujian Province, and the water resoureces are abundant in some Province. For example, water resources are abundant in Henan Province has over 1500 rivers, and annual rainfall is between 532 and 1380 mm (Henan province rainfall is between 532 and 1380 mm (Henan province 2015a, http://www.gov.cn/guoqing/2013-04/08/content_2583733.htm). In this region, rainfall occurs primarily from June to August and the annual average temperature is 12–19 °C. Thus, this region exhibits abundant rainfall and long periods of high-temperature weather, high temperatures and rainfall occurring in the same periods. Furthermore, if grain is not dried or remains exposed to high moisture levels during storage, these mycotoxins may occur. The hot and humid environment of this regeion is particularly suitable for mould growth, resulting in a serious mycotoxin contamination of grains. Therefore, different regions and different collected years may production of different values for mycotoxins. Meanwhile, different countries have different maximum admissible levels for AFB_1_, ZEN and DON [3, 14]. The United states Food and Drug Administration has set a maximum permissible level of 20 μg/kg for total aflatoxins in all foodstuff, while in China, the legal limit for AFB_1_ contamination in corn is 50 μg/kg (Table 4). The regulatory limit of AFB_1_, ZEN and DON in China and European regulatory limits were showed in Table 4. Pig are among the most sensitive species to all of these mycotoxins [2, 4, 5, 7–11]. The use of corn, wheat, DDGS and other feed ingredients for animal feeds increased the mycotoxins content in these feeds, resulting in fewer case of animal poisoning to exceeding to regulatory limit. Of course, the mycotoxins contaminated in animal feeds are an potential threat to human health, and the reduction of mycotoxins in contamination will requirement an integrated understanding of agronomy, fungal ecology, harvesting methods, storage conditions, feed processing and effective detoxification strategies for prevention public awareness [3, 6]. Moreover, the periodic surveillance and monitoring of the occurrence of mycotoxins in feed ingredients and complete feed are very important for human and animal health [14].

**Table 4:** Maximum limit regulations for aflatoxin B_1_ (AFB_1_), zearalenone (ZEN) and deoxynivalenol (DON) in feedstuff from China and European

Region	Mycotoxin	Feedstuff	Maximum level or guidance Value^c^, μg/kg	Reference standard
China	AFB_1_	Corn	50	GB^a^ 13078-2001
		Wheat	5.0	GB 2761-2011
		DDGS	50	NY/T^b^ 1968-2010
		Bran	40	GB 13078-2001
		Wheat shorts and red dog	40	GB 13078-2001
		Corn germ meal	50	GB 13078-2001
		Soybean meal	30	GB 13078-2001
		Pig complete feed	45	GB 13078-2001
		Duck complete feed	35	GB 13078-2001
		Cattle complete feed	45	GB 13078-2001
European	AFB_1_	Compound feed for dairy animals and young animals	5	European Parliament 2002
		Feed materials	20	European Parliament 2002
China	ZEN	Corn	500	GB 13078.2-2006
		Wheat	60	GB 2761-2011
		Complete feed	500	GB 13078.2-2006
European	ZEN	Complementary and complete feeding stuffs for piglets and gilts	100	European Commission 2006
		Complementary and complete feeding stuffs for calves, dairy cattle, sheep and goats	500	European Commission 2006
		maize by products	3000	European Commission 2006
China	DON	Corn	1000	GB 2761-2011
		Wheat	1000	GB 2761-2011
		DDGS	1000	NY/T 1968-2010
		Pig complete feed	1000	GB 13078.3-2007
European	DON	Complementary and complete feeding stuffs for pigs	900	European Commission 2006
		Cereals and cereal products	8000	European Commission 2006
		Maize by-products	12000	European Commission 2006

^a^GB: General Administration of Quality Supervision, Inspection and Quarantine of the People’s Republic of China. National Standard of the People’s Republic of China

^b^NY/T Ministry of Agriculture of the People’s Republic of China. Agricultural Standard of the People’s Republic of China

^c^moisture content: 12 %

## Conclusions

In conclusion, the detection results in our study showed that the content of all three mycotoxins contamination is a serious problem from different Province in China and differs among different years and that complete feed and feed ingredient are most often contaminated with DON, followed by ZEN and AFB_1_. The use of corn, domestic DDGS, imported DDGS and corn germ meal, which may be contaminated with these three mycotoxins, as animal feed may triggered a health risk for animal. More important, manufacturers should pay attention to the high levels of DON in corn and DDGS. In the future, mycotoxins contamination in feed and feed ingredients should be monitored routinely, and effective mycotoxins detoxification strategies should be selected according to realistic conditions.

## Abbreviations

AFB_1_: Aflatoxin B_1_
DDGS: Distillers dried grains with soluble
DON: Deoxynivalenol
ZEN: Zearalenone
